# Bridging the Gap: Integrating Knowledge from the Study of Social Network Analysis and Infectious Disease Dynamics in Human and Nonhuman Primates

**DOI:** 10.1146/annurev-anthro-052721-085447

**Published:** 2024-06-19

**Authors:** Jessica R. Deere, Elizabeth V. Lonsdorf, Julie A. Clennon, Thomas R. Gillespie

**Affiliations:** 1 Department of Environmental Sciences, Emory University, Atlanta, Georgia, USA;; 2 Department of Environmental Health, Rollins School of Public Health, Emory University, Atlanta, Georgia, USA; 3 Department of Anthropology, Emory University, Atlanta, Georgia, USA

**Keywords:** contact networks, One Health, public health, wildlife conservation, zoonoses

## Abstract

Primates live in complex social systems, and social contact and disease interact to shape the evolution of animal (including human) sociality. Researchers use social network analysis (SNA), a method of mapping and measuring contact patterns within a network of individuals, to understand the role that social interactions play in disease transmission. Here, we review lessons learned from SNA of humans and nonhuman primates (NHPs) and explore how they can inform health and wildlife conservation. Utilizing the breadth of knowledge in human systems and outlining how we can integrate that knowledge into our understanding of NHP sociality will add to our comprehension of disease transmission in NHP social networks and, in turn, will reveal more about human disease and well-being.

## 1. INTRODUCTION

Humans and nonhuman primates (NHPs) live in complex social systems that influence pathogen transmission within and between their populations (McCowan et al. 2016). Both humans and NHPs exhibit social structures and interpersonal relationships that create opportunities for pathogen spread (Funk et al. 2010, Griffin & Nunn 2012, Kappeler et al. 2015, Silk 2007). For humans, densely populated urban areas, global travel, and interconnected social networks contribute to the rapid dissemination of pathogens from local to global scales. NHPs, living in tightly knit social groups, experience similar dynamics on a local scale. Proximity between individuals, frequent physical contact, and shared resources facilitate transmission of infectious diseases. Understanding these complex and often dynamic interactions is crucial for managing and preventing disease outbreaks, as interventions must address not only the biology of pathogens but also the social and environmental factors that facilitate their transmission within human and NHP communities.

In NHPs, social behaviors, such as grooming, cooperation, and group living, have evolved as adaptive strategies to enhance protection against predators, acquire resources, and support collective caregiving (Gillespie & Chapman 2001). However, the proximity inherent in social structures also facilitates the transmission of pathogens (Balasubramaniam et al. 2019, Deere et al. 2021, Romano et al. 2020, Salathé et al. 2010). Pathogens, in turn, act as a selective pressure, driving the evolution of mechanisms that enhance resistance and tolerance (Ashby & Farine 2022, Poulin & Filion 2021, Udiani & Fefferman 2020). Social conditions shape the environment that individuals experience, and social adversity is closely linked to health and mortality outcomes (Snyder-Mackler et al. 2020). In many social mammal species, morbidity and mortality decrease as social advantages increase (Snyder-Mackler et al. 2020). In contrast, while social bonds are an integral part of group life and can benefit health (Ezenwa et al. 2016), sociality in humans and other animals comes at a cost through the increased risk of pathogen transmission due to proximity and contact. A better understanding of this trade-off between the benefits of sociality and the risks of disease transmission offers valuable insights into the evolution of social systems and the maintenance of health within populations.

The risk of zoonotic pathogen transmission, especially in systems characterized by high rates of human–NHP overlap (Bowden & Drake 2013, Pedersen et al. 2005, Plowright et al. 2017), highlights the importance of understanding how sociality impacts transmission. Anthropogenic change leads to more frequent contact between humans and NHPs, which results in an increased risk of cross-species transmission (Gibb et al. 2020, Gillespie et al. 2008, Nunn & Gillespie 2016, Shano et al. 2021). Humans are susceptible to pathogens that originate in NHPs, and, conversely, pathogen transmission from humans threatens NHPs. The close phylogenetic relationship between humans and NHPs further facilitates pathogen transmission across species (Calvignac-Spencer et al. 2012, Damas et al. 2020). Increasing our understanding of primate infectious disease dynamics is particularly pressing now, as pathogens have the potential to worsen the decline of already endangered primate populations (Estrada et al. 2017).

Knowledge of key biological processes, such as disease, has been greatly enhanced through the contributions of social network analysis (SNA), a method of mapping and measuring contact patterns within a network of individuals. The spread of pathogens fundamentally relies on contact among hosts, whether direct or indirect. Researchers use SNA to understand the role that social interactions play in disease transmission (Martínez-López et al. 2009) and to examine how social contact influences disease transmission in human and NHP populations. In this review, we aim to draw on lessons learned from the SNA of humans and NHPs. The history and methods of SNA have been reviewed thoroughly elsewhere (Brent et al. 2011, Croft et al. 2008, Dale & Fortin 2021, Danon et al. 2011, Freeman 2004, Keeling & Eames 2005, Luke & Harris 2007, Smith & Christakis 2008, Sueur et al. 2011, Wasserman & Faust 1994, Wey et al. 2008), so we do not intend to do that here. Instead, we focus on bridging the gap between human sociality and NHP network dynamics. Drawing from the breadth of knowledge in human systems and applying it to our understanding of NHP sociality will add to our comprehension of disease transmission in NHP social networks, while also offering insights into human disease and well-being.

## 2. SOCIAL NETWORK ANALYSIS AND DISEASE

**Figure 1:**
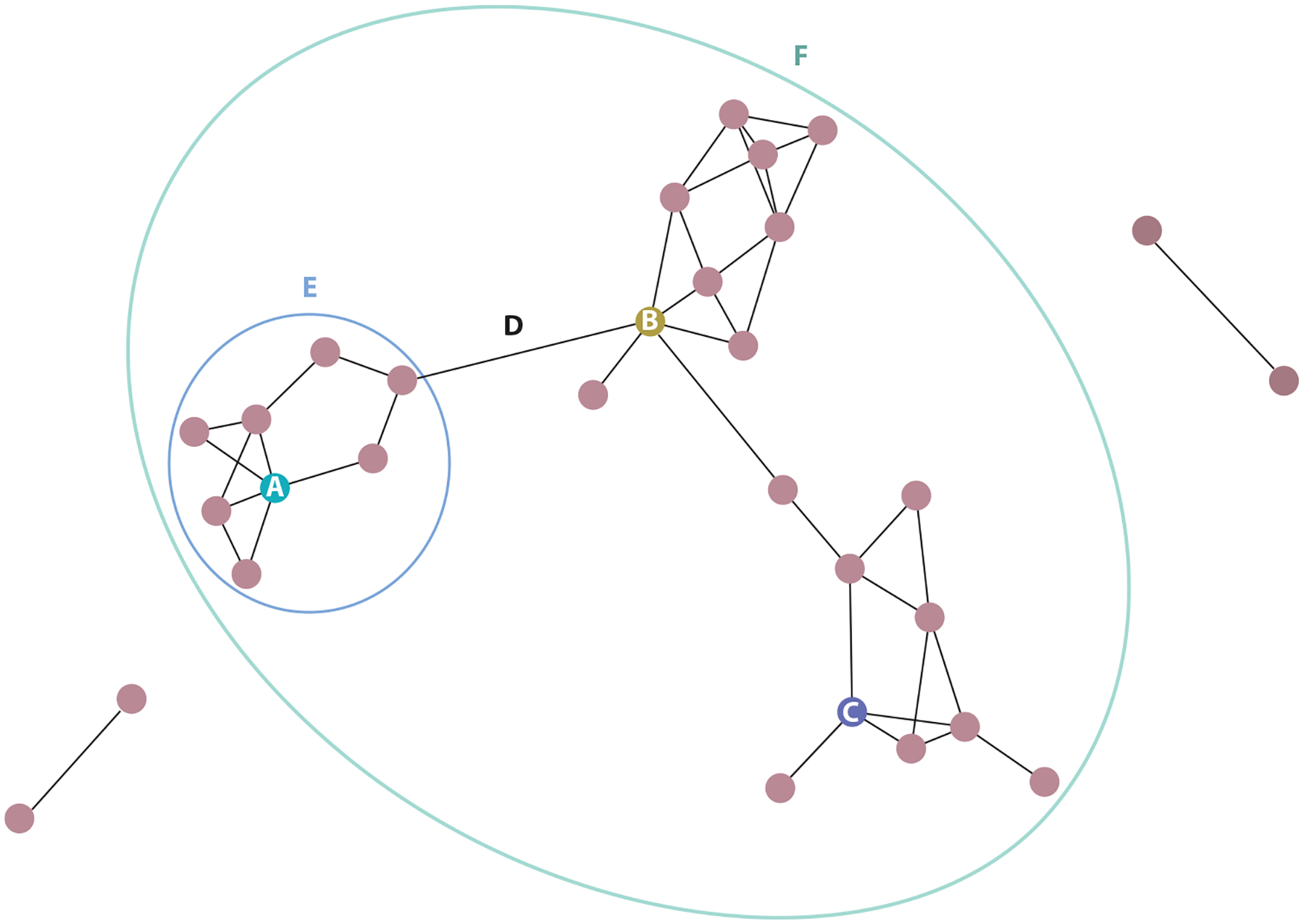
Basic elements of social network structure consist of nodes (A, B, and C), edges (D), modules (E), and components (F). Node A embodies high degree (i.e., node has many network connections). Node B embodies high betweenness (i.e., node is frequently along the shortest path between two other nodes). Node C embodies high closeness (i.e., node is closely connected to all other network nodes). Edges are the connection between two nodes and can be weighted by frequency or duration of interactions. Modules encompass a densely connected set of nodes. A component is a network zone disconnected from other parts of the network.

Public health infectious disease interventions use contact networks to identify cases and control pathogen spread through contact tracing (Luke & Harris 2007). This contact network approach can be used to explore epidemiological links among transmission pathways in NHP populations as well (Rushmore et al. 2017). A network consists of nodes (i.e., individuals) and edges (i.e., connections between individuals), and pathogens spread along edges according to who associates with whom (Figure 1). The impact of an individual’s network position on their susceptibility to infection is the key question for many studies of host–pathogen networks. Current literature consistently indicates that infection status is strongly influenced by centrality (i.e., level of connectedness within the network). Measures of centrality lie along a spectrum from local (e.g., degree, strength) to intermediate (e.g., eigenvector centrality) to global (e.g., closeness and betweenness) network position, each providing different insights into network characteristics (Farine & Whitehead 2015, Silk et al. 2017, Wey et al. 2008).

Most humans and NHPs live in social groups, creating subdivided networks. In such scenarios, local metrics are more effective at capturing short-term infection exposure when infected individuals are near, and global metrics are more effective for understanding an individual’s potential to spread a given infection (Silk et al. 2017). Documenting a complete contact network requires an understanding of the pathogen’s transmission route(s), sampling the targeted subpopulation, and capturing all interactions among sampled individuals that could result in infection (Sah et al. 2021). SNA integrates heterogeneity in interaction patterns at individual, local, and population scales to model the spread of infectious diseases (White et al. 2017). A key focus of network epidemiology is to identify what makes populations vulnerable to epidemics or any disease spread. Researchers use SNA to identify key individuals who play a significant role in transmission within communities.

SNA provides a valuable framework for addressing important questions related to infectious disease dynamics as well as disease monitoring, the examination of disease research, and the assessment of communication strategies in disease programs. How network position affects infection susceptibility is one such inquiry. We can use SNA to quantify the risk associated with specific nodes, which identifies individuals at greater vulnerability. This approach also enables the identification of those with disproportionately high contact levels: superspreaders (see the sidebar titled Superspreaders). Targeting these influential nodes for vaccination, treatment, or isolation can enhance disease control strategies (Lloyd-Smith et al. 2005). Furthermore, SNA offers insights into how overall network structure influences infection patterns (e.g., prevalence, basic reproductive number, rate of spread). This methodology allows us to quantify population vulnerability over time and design more realistic models of disease spread. By incorporating network structure into disease management strategies, we can develop more targeted surveillance and control measures, thus enhancing our ability to respond effectively to disease threats.

### 2.1. SNA and Infectious Disease in Humans: A Brief History

While modern SNA is rooted in sociology through the works of Jacob Moreno in the 1930s (Moreno 1934, Moreno & Jennings 1938) and Harrison White in the early 1970s (Freeman 2004, Wasserman & Faust 1994), the application of SNA to understand the spread and dynamics of infectious diseases gained prominence in the late twentieth century. SNA relies on graph theory (i.e., studying relationships between objects) to analyze and understand the structure, dynamics, and properties of social networks (Freeman 2004, Wasserman & Faust 1994). With the advent of sophisticated data collection and analysis methods, SNA has been applied to study the spread of various infectious diseases.

SNA gained momentum in epidemiological studies in the 1980s to study transmission patterns and social dynamics influencing HIV spread (Altmann et al. 1994, Klovdahl 1985). SNA has been widely used to study the transmission of other sexually transmitted infections (STIs) (Danon et al. 2011). Researchers examine contact networks to understand how STIs spread through populations and identify factors contributing to high-risk behavior (Wylie et al. 2005). SNA is also used to optimize vaccination strategies. Identifying key individuals or groups within a social network can help target vaccination campaigns to achieve maximum impact (Saunders & Schwartz 2021). SNA has also been instrumental in designing and evaluating behavioral interventions to prevent pathogen spread and improve public health (Shelton et al. 2019). Researchers can tailor interventions to target influential individuals or groups within a community by understanding social networks.

While early research in network epidemiology studied HIV/AIDS and STIs (Luke & Harris 2007), the application of SNA has expanded to tackle influenza (Cauchemez et al. 2011), tuberculosis (Gardy et al. 2011), severe acute respiratory syndrome (SARS) (Meyers et al. 2005), and COVID-19 (Block et al. 2020, Firth et al. 2020). These examples highlight the diverse applications of SNA in disease studies. The field continues to evolve as technology and data analysis techniques advance, allowing for more sophisticated and nuanced investigations into social aspects of disease transmission.

### 2.2. SNA and Infectious Disease in NHPs: A Brief History

While SNA initially focused on the dynamics of human interactions, integration of SNA into studies of NHP social behavior began in the 1960s (Sade 1965, 1972). Over the past two decades, primatologists have expanded their use of SNA (Brent et al. 2011, Puga-Gonzalez et al. 2019), with a tenfold increase in SNA applied to primate behavioral studies (Puga-Gonzalez et al. 2019) and primate–pathogen network studies (Rushmore et al. 2017). Most primate–pathogen network studies have (*a*) employed models to simulate pathogen dissemination within empirical contact networks (Romano et al. 2016, Rushmore et al. 2014), (*b*) integrated empirical data on social contact and infection status in natural systems (Deere et al. 2021, Rimbach et al. 2015), or (*c*) compared networks across species to discern interspecific variation (Gómez et al. 2013, Rushmore et al. 2017).

As in human health research, primatologists use SNA to identify individuals or groups within NHP populations that play important roles in disease transmission. Researchers discover central individuals who may serve as superspreaders by analyzing social networks (Carne et al. 2013). Insights from SNA studies inform the development of targeted interventions and wildlife conservation strategies to manage and mitigate disease risks in NHP populations. This practice may involve targeting specific individuals for vaccination, a crucial approach when challenges exist in vaccinating an entire population due to logistical constraints (Rushmore et al. 2014). SNA guides our understanding of contact patterns within primate groups, including affiliative interactions, grooming networks, and spatial associations. This information is vital for assessing disease transmission risks and understanding disease spread dynamics. Studies using SNA have provided valuable insights into how social structures influence disease spread in NHP populations, including understanding how social network structure affects transmission rate and the potential for localized or widespread outbreaks. SNA is also used to investigate social connectivity and physiological predictors of pathogen acquisition. For example, SNA was used to measure the risk of reinfection of helminths after antiparasitic treatment (Friant et al. 2016).

Researchers have also used SNA to investigate how environmental factors, such as habitat fragmentation and human–wildlife interactions, can influence disease dynamics in NHPs. Human-dominated landscapes have changed social networks in ways that are important for disease transmission, such as increasing overlap between humans and NHPs or altering grouping patterns (Balasubramaniam et al. 2021, Satsias et al. 2022). SNA has facilitated comparative studies across different NHP species (Carne et al. 2013, 2014; Griffin & Nunn 2012) and between humans and NHPs (Balasubramaniam et al. 2021, Gómez et al. 2013). By analyzing social networks in various species, researchers can identify similarities and differences in disease dynamics, contributing to a broader understanding of the ecological and evolutionary factors at play. Advances in technology, including the use of proximity loggers and high-resolution global positioning system (GPS) collars (Strandburg-Peshkin et al. 2015), have enhanced the accuracy and depth of data used in SNA studies. The history of SNA and disease in NHPs reflects a growing recognition of the importance of social structures in shaping disease dynamics, which advances our understanding of how diseases spread within NHP populations and has implications for wildlife conservation and public health.

## 3. LESSONS LEARNED: SOCIAL NETWORK ANALYSIS AND DISEASE IN HUMANS

The COVID-19 pandemic painfully demonstrated the importance of understanding how the dynamics of health-related behaviors affect pathogen spread. SNA was used to model and analyze transmission dynamics during the pandemic (Nande et al. 2021, Yang 2021). By studying connections among individuals, researchers identified high-risk clusters, uncovered how the virus spread through social networks, and determined methods for mitigating severe acute respiratory syndrome coronavirus 2 (SARS-CoV-2) spread (Block et al. 2020). SNA confirmed superspreading as important for SARS-CoV-2 spread and highlighted characteristics of such events, allowing for targeted preventive measures (Bouffanais & Lim 2020).

SNA is often used for contact tracing efforts during disease outbreaks and transmission events (Chen et al. 2011, Eames & Keeling 2003, Vazquez-Prokopec et al. 2017), including the COVID-19 pandemic (Firth et al. 2020, Nagarajan et al. 2020, Ostovari et al. 2020). Vigilant monitoring of those exposed to SARS-CoV-2 is crucial to promptly isolate these individuals, prevent potential transmission to secondary contacts, and ensure supportive care. By mapping social networks, researchers and public health officials identified and traced contacts of SARS-CoV-2-positive individuals (Jo et al. 2021), improving our understanding of potential transmission pathways and guiding targeted interventions (Hoang et al. 2022). However, the significant role played by asymptomatic individuals in SARS-CoV-2 transmission means that relying solely on manual contact tracing may not be adequately effective to contain spread across various outbreak scenarios (Nande et al. 2021). SNA determined that physical distancing combined with contact tracing was most effective at controlling SARS-CoV-2 outbreaks (Firth et al. 2020). That individuals with SARS-CoV-2 could spread the virus before showing symptoms highlighted the challenges of identifying and isolating cases promptly.

Beyond transmission dynamics, SNA provided insights on the effects of social and behavioral factors on SARS-CoV-2 spread, including how information about the virus was disseminated through social networks, including social media (Hung et al. 2020), and the influence of social conventions on adherence to preventive measures, such as vaccines. As vaccines became available, researchers used SNA to optimize vaccine distribution strategies (Saunders & Schwartz 2021). By identifying key individuals or groups within social networks, public health officials could prioritize vaccination efforts to maximally reduce transmission. Identifying key populations for vaccination, addressing vaccine hesitancy, and ensuring equitable distribution were all aspects that benefited from insights into social networks and health-related behaviors. The COVID-19 pandemic brought to the forefront the necessity of understanding the spread of infectious diseases and the interplay of health-related behaviors, including social networks.

Beyond COVID-19, many studies—which have focused on specific pathogens such as HIV (Christley et al. 2005), mpox (formerly known as monkeypox) virus (Endo et al. 2022), and tuberculosis (Gardy et al. 2011)—incorporate network structures to understand transmission dynamics. In epidemiology, various network types can be examined, such as social or sexual contacts, patient movement between hospitals, or air travel between cities (Eames et al. 2015). To gather information related to these networks, three primary methods have been used: infection tracing, complete contact tracing, and diary-based studies (Keeling & Eames 2005, Read et al. 2008). The choice among methods depends on available resources and specific objectives.

Digital epidemiology and computational methods have enhanced our understanding of human disease transmission (Drewe & Perkins 2015, Salathé et al. 2012). When integrated with SNA, digital epidemiology provides a powerful tool for tracing contacts and interactions of infected individuals. Digital epidemiology leverages digital technologies and data sources—including social media, online search queries, mobile phone records, and wearable devices—to monitor, model, and analyze disease spread in real time (Salathé et al. 2012). Large datasets (i.e., big data) are often associated with digital epidemiology, which comes with both advantages and limitations. While they are likely to provide a larger sample size from humans compared with social networks of NHPs, data from sources such as mobile phones and social media are not always a good proxy for contact data and do not include the infection status of tracked individuals (Drewe & Perkins 2015). We can also use SNA for information transmission, as digital epidemiology can gauge public sentiment, attitudes, and compliance with preventive measures during outbreaks (Allington et al. 2021, Coiera 2013, Hung et al. 2020). SNA can identify key opinion leaders and influential voices that can impact public behavior (Zhu et al. 2020). Information transmission networks are essential for understanding how health-related information spreads, influences behavior, and impacts disease transmission. These networks help shape public health strategies and aid in dissemination of health-related information (Hung et al. 2020, Kawonga et al. 2015, Luke & Harris 2007). By harnessing digital data sources and computational methods, researchers can gain valuable insights into the complex interplay of social connections, behaviors, and infectious diseases.

## 4. LESSONS LEARNED: SOCIAL NETWORK ANALYSIS AND DISEASE IN NONHUMAN PRIMATES

SNA has also proven to be a valuable tool for understanding NHP transmission dynamics, providing critical insights into pathogen spread among our closest relatives. Despite only one known wild primate infection with SARS-CoV-2 (Pereira et al. 2022), NHPs are broadly susceptible (Lu et al. 2020) and have homologous cellular receptor proteins for the virus (Damas et al. 2020), and some experience morbidity after being unintentionally infected in captivity (Tchesnokova et al. 2021). Thus, SNA should be employed to assess the risk of spillover and spillback events and guide measures to minimize human–NHP interactions that could lead to SARS-CoV-2 and other pathogen transmission (Fagre et al. 2022).

A primary lesson from SNA in NHPs is the profound impact of social structure on disease dynamics. Not unlike humans, most NHPs are highly social, and even less social species interact in ways that might facilitate disease transmission. For example, chimpanzees live in multimale/multifemale communities, characterized by fission-fusion dynamics with subgroup size and composition that vary throughout the day (Boesch & Boesch-Achermann 2000, Goodall 1986, Nishida 1968). Orangutans also live in fission-fusion social systems with fluid relationships and individuals assembling in temporary parties that regularly change in composition (Van Schaik 1999). However, orangutans are far less gregarious than chimpanzees, and thus chimpanzees and orangutans represent opposite ends of the sociality spectrum among apes. Gregariousness is associated with both costs and benefits of sociality in group-living animals, and the risk of pathogen transmission is often one of the costs of gregariousness (Côté & Poulin 1995, Patterson & Ruckstuhl 2013). SNA showed that gregariousness was associated with parasite infections in a community of wild chimpanzees, where chimpanzees who spent more time with more individuals in the same space had higher parasite species richness (Deere et al. 2021). Varying social dynamics among different NHP species provides an excellent opportunity to investigate how sociality influences pathogen transmission. SNA helps elucidate how these social structures influence disease transmission patterns and individuals’ susceptibility (Rushmore et al. 2013).

As observed in humans, SNA has revealed central individuals within NHP social networks (MacIntosh et al. 2012) and the role that highly connected individuals play in pathogen transmission dynamics (Rimbach et al. 2015). In areas where humans and NHPs coexist, SNA has been instrumental in studying the interface between these populations and their potential for disease transmission (Balasubramaniam et al. 2021). Both human and NHP studies employ network-based interventions, but the nature of interventions varies. In human populations, interventions involve vaccination campaigns, contact tracing, and behavior change communication. In NHPs, interventions include habitat protection, research guidelines, and wildlife management strategies. However, network-based vaccination has been considered for NHPs. Rushmore et al. (2014) demonstrated how SNA can be employed to optimize targeted and efficient vaccination among wild chimpanzees.

While some have proposed NHP social systems as valuable models for understanding human health, there has been limited discussion of how human social networks can be applied to benefit NHPs (McCowan et al. 2016). Human social networks exhibit social structures resembling those of other primates, and these structured networks significantly influence pathogen transmission rates. Moreover, anthropogenic changes are resulting in increased interactions and overlap between humans and NHPs. A recent study used SNA to investigate how wild chimpanzees in Uganda adjusted their social behavior in response to human-modified landscapes (Satsias et al. 2022). These chimpanzees frequently adjusted social grouping dynamics and social behavior in response to perceived anthropogenic risks, with notable distinctions between males and females (Satsias et al. 2022). Factors that result from changes in social dynamics induced by anthropogenic impacts—such as limited access to natural resources, increased opportunities for interspecies and conspecific overlap, and altered life-history strategies—have the potential to impact ecological and evolutionary processes influenced by social structure, including pathogen spread.

SNA can inform wildlife conservation strategies (Snijders et al. 2017), especially in the context of NHPs vulnerable to disease outbreaks. This data-driven approach can guide decisions related to habitat preservation and wildlife management (Carne et al. 2013, Rushmore et al. 2017). For example, Anise et al. (2024) used SNA to retrospectively investigate when northern muriquis, an endangered primate, began to show signs of group fission, providing important implications for conservation management in a fragmented habitat. By examining social networks of endangered species, researchers can identify key individuals for protection and intervention, ensuring the preservation of genetic diversity and long-term population viability.

## 5. FUTURE DIRECTIONS

In the face of a growing human population and heightened interactions and contact rates across primate habitats, we must urgently improve our understanding of how anthropogenic activities alter wild primate contact networks with humans and our domesticated animals and associated disease-related risks (Gillespie et al. 2021). For example, when research or tourism is initiated, primates are acclimated to human presence through a process known as habituation. An unintended consequence of habituation is exposure to human pathogens (Gilardi et al. 2015), with even mild human pathogens causing moderate-to-severe outcomes in wild primates (Köndgen et al. 2008, Negrey et al. 2019, Palacios et al. 2011, Patrono et al. 2018). Expansion of commercial or subsistence agriculture into primate habitat has similarly facilitated primate exposure to the pathogens of people and domesticated animals (Bublitz et al. 2015, Johnston et al. 2010, Ragazzo et al. 2018, Salyer et al. 2012). NHPs are already threatened by habitat loss, hunting, and climate change, so the introduction of human pathogens has the potential to lead to primate extinctions (Estrada et al. 2017, Gillespie & Leendertz 2020). To best guide primate conservation and management, and to protect people who live near NHPs, we need to better understand how social contact shapes disease spread in humans and NHPs.

Infectious disease dynamics have historically been represented through compartmental models—a mathematical approach to describe and analyze complex systems by dividing them into interconnected compartments or regions—that assume a homogeneous mixing of individuals (Anderson & May 1991); however, humans and NHPs often interact nonrandomly. Thus, models that assume heterogeneity in number and duration of contacts will be more realistic, capturing sociological, temporal, and/or spatial clustering of contacts. SNA offers a distinct advantage over traditional compartmental modeling by incorporating heterogeneity in interaction patterns across individual, local, and population levels, enabling modeling of global-level processes. This approach includes the dissemination of social information and transmission of infectious diseases, providing a comprehensive understanding of interconnected dynamics.

For research in both humans and NHPs, SNA and compartmental modeling can be combined by developing models of transmission through networks. However, we need to know the presence and absence of a contact and the weight, duration, and timing of occurrence. Thus, gathering all needed information comes with challenges. Eames et al. (2015) laid out six challenges for measuring empirical contact networks relevant to infectious disease modeling: (*a*) defining contacts; (*b*) confining a network to relevant space, time, and scope; (*c*) accounting for missing data; (*d*) measuring nonstatic networks (i.e., weighted, dynamic); (*e*) making use of indirect network information; and (*f*) privacy and ethical concerns. These challenges apply to both human and NHP networks and should be considered when using SNA to investigate disease transmission.

Knowledge of network structure, combined with theoretical or empirical knowledge of pathogen transmission dynamics, allows us to estimate population-scale epidemic dynamics from individual-level interactions (Keeling & Eames 2005). Integration of epidemiological compartmental models with network models is particularly relevant for studying zoonotic disease transmission dynamics, where understanding human–NHP interactions is crucial for effective disease control. For studies aiming to capture the complexity of interactions among individuals and resulting impacts on pathogen spread, incorporating network structures is valuable for assessing the effectiveness of different intervention strategies (Vazquez-Prokopec et al. 2013, Volz et al. 2011). We can model the impact of targeted interventions on specific nodes or edges within networks to identify optimal control measures. Combining compartmental models with network models also allows for exploration of spatial and temporal dynamics of disease spread. Understanding how individuals move through space and time can provide deeper insights into the spatial patterns of epidemics.

**Figure 2:**
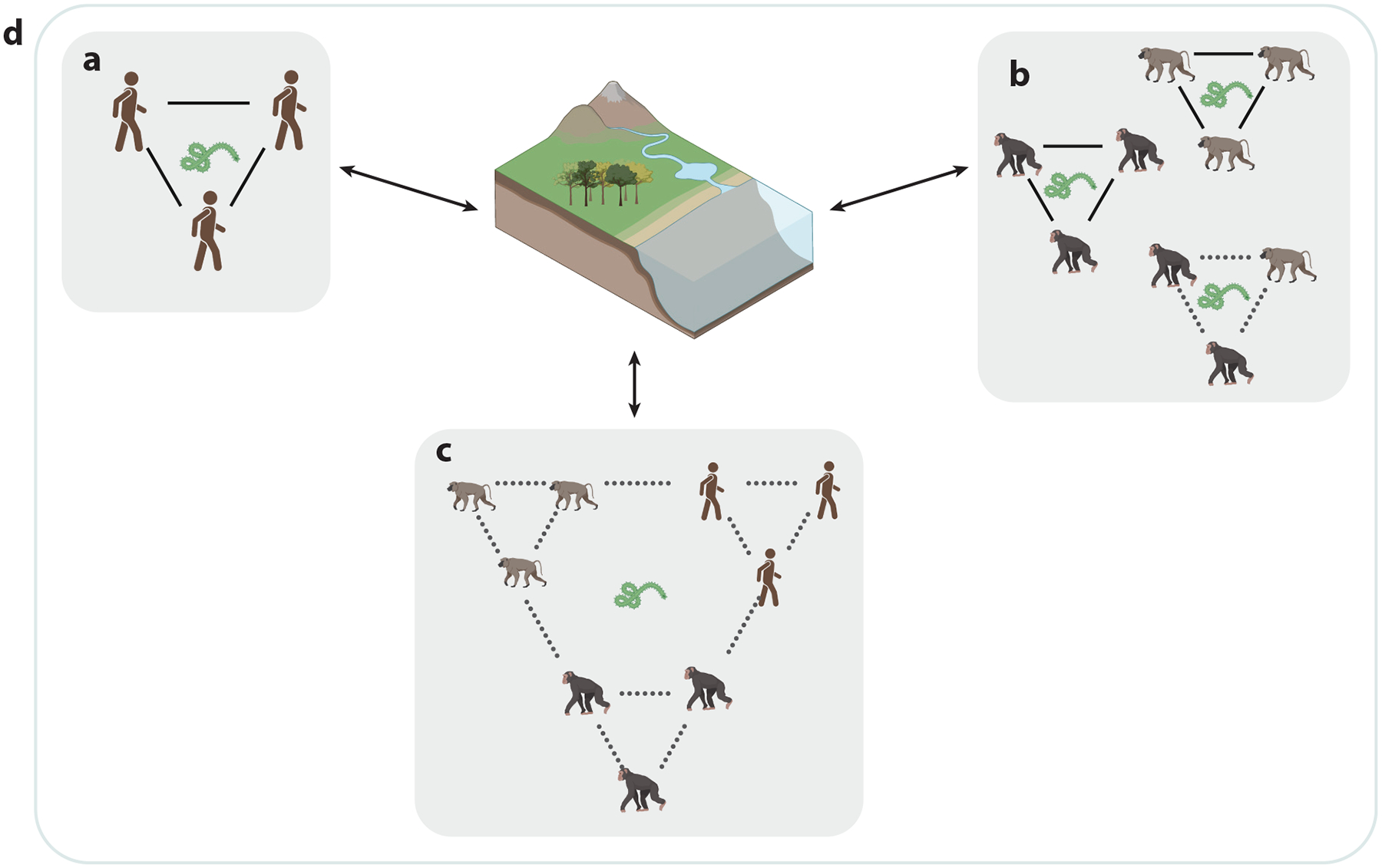
Conceptual diagram of human social network (*a*); three nonhuman primate social networks (*b*); co-occurrence network including humans, apes, and monkeys (*c*); and a shared spatiotemporal environment where all networks interact (*d*). Dotted lines (i.e., edges) represent multiple-species networks, and solid lines represent single-species networks. Arrows represent integration of multispecies and spatiotemporal networks. The relationship between networks and environment is bidirectional, as one’s network position is intertwined with their spatial behavior, while, simultaneously, social environment can influence one’s space-use decisions. Zoonotic pathogens, such as Ebola virus (pictured as an example), can be transmitted through any of these network pathways, but many pathogens rely on other environmental factors in addition to social contact events for transmission. Thus, quantifying space and sociality simultaneously is important. Figure created with BioRender.com.

The escalating frequency and expansion of interactions between humans and NHPs underscore the need for co-occurrence networks, which assess spatiotemporal associations between humans and NHPs (Figure 2). These networks allow us to navigate complex connections, track patterns of co-occurrence, and gain insights into the potential for information exchange and zoonotic spillover and spillback events. By mapping co-occurrence of humans and NHPs in shared environments, we can identify potential hot spots for disease spillover and assess the risk of cross-species transmission. SNA was implemented to study co-occurrence of humans and three macaque species, identifying some macaques as more central in their human co-interaction networks, potentially serving as superspreaders for zoonotic disease transmission (Balasubramaniam et al. 2021). More systematic collection of disease data such as these would facilitate proactive measures to mitigate pathogen spread to protect humans and NHPs.

Co-occurrence networks can elucidate spatiotemporal patterns and frequency of interactions between NHPs and humans, which can guide targeted interventions aimed at preventing disease transmission. Co-occurrence networks can serve as a valuable tool for forming effective strategies to safeguard public health and the conservation of NHPs. Identifying patterns of co-occurrence of humans and NHPs and their interactions within specific geographic areas is an important factor in monitoring zoonotic diseases because detecting co-occurrence patterns can serve as an early warning system for potential disease outbreaks.

The incorporation of spatial analysis into SNA (i.e., spatial-social analysis) will help elucidate disease dynamics within primate social networks by increasing the scope of disease transmission studies and allowing more accuracy and refinement (Albery et al. 2021). Scaling analyses up by incorporating spatiotemporal and co-occurrence data with traditional SNA will help address complex health-related challenges that involve One Health, the interconnectedness of human, animal, and environmental health (OHHLEP et al. 2022). Some models are designed to allow for changes within a network, but this adaptability is not standard and continues to represent an important challenge. By tracking the movements and interactions of individuals in real time, researchers can capture dynamic changes in social networks. These data can reveal how interactions fluctuate with seasonality, migration, or other environmental factors, providing insights into disease spread over time.

Data-logging devices, such as GPS and proximity loggers, are often used in research to track movements and interactions of humans and animals (Albery et al. 2021; Gilbertson et al. 2021; Parsons et al. 2014; Vazquez-Prokopec et al. 2009, 2013). While some have successfully tracked NHPs with GPS collars (Strandburg-Peshkin et al. 2015), fitting NHPs with GPS collars comes with ethical concerns and logistical challenges. Fortunately, technological advances in motion-tracking cameras and camera traps hold promise for integration with traditional behavioral observations for comprehensive and safe data collection. Nevertheless, GPS data allow for identification of high-risk areas where humans, NHPs, and other animals overlap (Parsons et al. 2014). These locations can serve as potential hot spots for disease transmission, helping prioritize surveillance and intervention efforts. Simultaneous tracking of humans, NHPs, and other animals via GPS data can also inform predictive models of disease transmission. By combining movement patterns, social interactions, and environmental variables, researchers can develop models that forecast outbreak risks and inform prevention strategies. GPS tracking and disease studies can aid in conservation efforts by monitoring the movements and habitats of wild primates and their interactions with human activities, which can inform wildlife management and conservation strategies (Lonsdorf et al. 2022).

Incorporating genomic methods and molecular epidemiology into SNA to study infectious diseases enhances our ability to understand disease transmission pathways (Gilbertson et al. 2018, Vasylyeva et al. 2016). In addition, the use of multilayer networks makes analyses more realistic when there is more than one type of transmission-relevant interaction (Kinsley et al. 2020). Multilevel approaches capture heterogeneity in contact patterns across different spatial and temporal layers. Furthermore, advancements in computational power and development of SNA-specific software programs and packages will lead to new discoveries. These advanced, multidisciplinary approaches will offer valuable insights that can inform public health strategies, improve outbreak response, and conserve wildlife.

## 6. CONCLUSION

SNA has emerged as a powerful tool in understanding the dynamics of disease transmission in human and NHP populations. SNA offers a natural framework for comprehending how contact patterns influence pathogen dynamics, as the frequency of contact is central to pathogen transmission. These connections among individuals or groups, which facilitate pathogen spread, inherently constitute a network. This network, in turn, yields valuable insights into epidemiologic dynamics at play. Over time, researchers have conducted extensive studies to investigate how social networks impact the spread of infectious diseases, and these studies have yielded significant findings that have contributed to our understanding of public health and wildlife epidemiology. This field is dynamic, and ongoing research continues to refine and expand our understanding of how contact among individuals and between species influences pathogen spread.

The study of both human and NHP social networks and their role in disease transmission allows us to gain a comprehensive understanding of how infectious diseases spread across species boundaries, with significant implications for public health and wildlife conservation. By examining social structures, interactions, and disease dynamics between humans and NHPs, we can identify potential sources of zoonotic infections and assess the risk of disease spillover. This interdisciplinary research fosters collaboration among fields such as epidemiology, primatology, ecology, and anthropology, enabling a more holistic approach to disease control and prevention. It highlights the interconnectedness of human, animal, and environmental health, aligning with the One Health perspective.

The insights gained from studying social networks can inform targeted interventions, facilitate early warning systems, and guide strategies to mitigate disease spread. By identifying key influencers, superspreaders, and critical nodes in these networks, we can tailor public health measures and conservation efforts for better effectiveness. Linking the dialogue between studies on humans and studies on NHPs regarding the role of sociality in influencing disease transmission enhances our understanding of disease ecology and evolution in humans and NHPs.

## SUPERSPREADERS

Superspreading occurs when a small number of individuals infect an unusually large number of individuals during an infectious disease outbreak (Lloyd-Smith et al. 2005). These highly infectious individuals play a disproportionate role in pathogen transmission. Superspreading events arise when a single individual engaging in close contact with others leads to rapid and widespread transmission of a pathogen. Superspreaders are often identified through SNA as nodes with high degrees of connectivity or centrality. For example, individuals with high betweenness, those important in connecting to all individuals in a network, are likely to be superspreaders during epidemics. Superspreading introduces heterogeneity into network structure (Vanderwaal & Ezenwa 2016). While some nodes have minimal impact on disease transmission, superspreaders have the potential to cause large outbreaks due to their extensive connections. Greater heterogeneity in degree distribution increases the probability of superspreading events occurring if a pathogen reemerges (Silk & Fefferman 2021). The 20/80 rule (20% of cases cause 80% of transmission) often applies during superspreading events, allowing for the identification of high-risk individuals (Woolhouse et al. 1997). By focusing on these key individuals, public health and wildlife conservation measures can be more efficiently deployed to mitigate disease impact.

## Abbreviations

Contact tracing: identifying individuals who have a contact history with infected individuals
Node: an individual in a network
Edge: connection between two nodes in a network indicating a relationship between them
Centrality: a node’s importance within a network
Degree: the number of edges connecting one node to other nodes in the network
Strength: summed weight of all adjacent connections for each node in a network
Eigenvector centrality: network influence of a node when second-order connections are considered
Closeness: measure of how close a node is to all other nodes in a network
Betweenness: number of times a node is on the shortest path between two other nodes in a network
Local metrics: measures that take into account only immediate network neighbors
Global metrics: measures that take entire network structure into account
Superspreader: an individual who plays a disproportionate role in the spread of a pathogen
Basic reproductive number: average number of new infections caused by one infected individual in a fully susceptible population
Spillover: transmission of disease from animals to humans
Spillback: transmission of disease from humans to animals
Zoonotic disease: a pathogen transmitted between humans and animals
